# The role of dental status in the pathogenesis and severity of peritonsillar and cervical infections

**DOI:** 10.3389/fmed.2025.1590310

**Published:** 2025-07-14

**Authors:** Florian Ciprian Venter, Timea Claudia Ghitea, Adrian Nicolae Venter, Amin-Florin El-Kharoubi, Mousa El-Kharoubi, Evelin Claudia Ghitea, Marc Cristian Ghitea, Amina Venter

**Affiliations:** ^1^Doctoral School of Biological and Biomedical Sciences, University of Oradea, Oradea, Romania; ^2^Department of Pharmacy, Faculty of Medicine and Pharmacy, University of Oradea, Oradea, Romania; ^3^Bihor Clinical County Emergency Hospital, Oradea, Romania; ^4^The County Emergency Clinical Hospital of Târgu Mureş, Târgu Mureş, Romania; ^5^Faculty of Medicine and Pharmacy, University of Oradea, Oradea, Romania

**Keywords:** peritonsillar infections, cervical abscesses, dental status, oral hygiene, dental work, dental implants, poor dentition, antibiotic therapy

## Abstract

**Background:**

Peritonsillar and cervical infections, such as peritonsillar phlegmon and laterocervical abscesses, are serious complications of oropharyngeal infections. Although poor dental status and inadequate oral hygiene are recognized risk factors, their association with infection severity remains insufficiently explored. This study aimed to assess the relationship between dental status and the clinical severity and management of peritonsillar and cervical infections.

**Materials and methods:**

In this retrospective cohort study, hospitalized patients diagnosed with peritonsillar phlegmon, laterocervical/submandibular abscess, or peritonsillar abscess were included. Clinical and biological data, including dental work, dental implants, dentition quality, treatment, and hospitalization duration, were analyzed. Statistical tests and regression analyses were performed to explore associations between dental status and infection-related outcomes.

**Results:**

Patients with prior dental work or compromised dentition more frequently underwent complex treatments, including corticosteroids. Absence of dental implants was associated with increased antibiotic use and prolonged hospitalization. Although patients with poor dentition required corticosteroids less frequently, they presented more severe infection patterns.

**Conclusion:**

Dental status may be associated with differences in infection severity and treatment patterns among patients with peritonsillar and cervical infections. These findings underscore the potential value of maintaining good oral health as part of a multidisciplinary approach involving ENT specialists and dental professionals.

## 1 Introduction

Peritonsillar and deep cervical infections represent significant clinical challenges due to their rapid progression and potential for severe complications (1). These infections, including peritonsillar abscess, phlegmon (2), and laterocervical or submandibular abscesses, often arise from local bacterial spread within the oropharyngeal and neck spaces (3). While upper respiratory tract infections are common precursors, growing evidence suggests that poor oral health may also contribute to their development and severity (4, 5).

Dental conditions such as untreated caries, periodontitis, apical abscesses, and poor dentition can act as microbial reservoirs for pathogenic bacteria capable of infiltrating deeper cervical structures. Inadequate oral hygiene facilitates bacterial plaque accumulation and dysbiosis of the oral microbiota, potentially increasing the risk of localized and systemic infections (6). The oral cavity, being a primary entry point to the gastrointestinal and respiratory tracts, is frequently exposed to pathogens that may migrate under favorable conditions, especially when the local or systemic immune defense is impaired (5).

Previous studies have reported that odontogenic infections constitute a substantial proportion of deep neck space infections. Furthermore, patients with poor oral hygiene and reduced access to dental care—often observed in rural populations—may have a higher incidence of complications due to delayed intervention or unrecognized dental sources of infection (7–9). Despite these observations, the specific association between dental status indicators (e.g., presence of dental work, implants, or tooth loss) and the clinical course of peritonsillar and cervical infections remains insufficiently investigated (10, 11).

The current body of literature emphasizes the importance of integrating oral health into broader medical care, particularly for conditions with an infectious or inflammatory component (12). However, few studies have systematically analyzed how dental status correlates with infection severity, treatment requirements, or clinical outcomes in peritonsillar and deep cervical infections. In clinical practice, dental factors are often overlooked in the management of oropharyngeal infections, despite their potential relevance to prognosis and recovery.

The primary objective of this retrospective cohort study is to assess whether various aspects of dental status are associated with differences in clinical presentation, therapeutic approach, and hospitalization duration among patients with peritonsillar and cervical infections. By analyzing patient records from a tertiary care hospital, we aim to identify oral health-related patterns that may inform a more interdisciplinary strategy for managing these infections. We hypothesize that poor dental status may correspond to more severe infection parameters and greater healthcare resource utilization.

## 2 Materials and methods

### 2.1 Study design and patient selection

This retrospective cohort study was conducted on a cohort of patients hospitalized with peritonsillar and cervical infections in a specialized medical unit. Data were extracted from patients’ medical records, analyzing clinical, biological, and imaging variables relevant to the assessment of dental status and infection severity. The study was conducted at the Emergency County Clinical Hospital of Oradea between January 2020 and December 2023.

Sample Size Considerations: As a retrospective study, we included all eligible patients meeting the criteria during the study period; no formal *a priori* sample size calculation was performed.

### 2.2 Inclusion and exclusion criteria

In total 108 patients were classified into three groups based on the type of diagnosed infection:

Group I: Patients with peritonsillar phlegmon, a diffuse infection of the soft tissues surrounding the tonsils, characterized by extensive inflammation and an increased risk of complications.

Group II: Patients with laterocervical/submandibular abscess, a deep infection affecting the cervical and submandibular spaces, with potential extension to critical neck structures.

Group III: Patients with peritonsillar abscess, a localized accumulation of pus between the tonsillar capsule and adjacent tissues, representing one of the most severe complications of untreated tonsillitis.

Inclusion Criteria:

Patients with clinically and imaging-confirmed peritonsillar and cervical infections.

Patients with complete records of dental history and previous treatments.

Patients aged 18 years or older.

Exclusion Criteria:

Patients with infections of non-dental origin.

Cases with incomplete medical records or missing dental history.

Severely immunocompromised patients or those with advanced oncological conditions.

### 2.3 Data collection and parameters analyzed

For each patient, the following variables were recorded and analyzed:

Demographics: Age, sex, and place of residence.

Dental Status: Presence of poor dentition, dental implants, or prosthetic dental work (as presence of prosthetic restorations, crowns, bridges, or fillings as recorded in the patient’s dental history). Normal dentition was defined as the presence of 24 or more natural teeth, without signs of decay, mobility, or periodontal disease, based on documented dental evaluation at admission or prior consultations.

Comorbidities: Diabetes mellitus, hypertension, chronic heart failure, liver diseases.

Inflammatory Markers: Leukocyte count, C-reactive protein (CRP), erythrocyte sedimentation rate (ESR).

Need for Surgical Intervention: Incision and drainage or other surgical procedures.

Length of Hospitalization: Measured in days.

Treatment Administered: Antibiotic therapy, corticosteroid therapy, and analgesic use.

### 2.4 Statistical analysis

Data were analyzed using specialized statistical software (SPSS, R). Group comparisons were performed using Student’s *t*-test for continuous variables and the Chi-square test for categorical variables. Correlations between dental status and infection severity were evaluated using logistic regression models, adjusting for relevant confounding variables. A significance level of *p* < 0.05 was considered statistically significant.

### 2.5 Ethical considerations

The study was approved by the Ethics Committee of the medical institution (Approval No. 9410/08.04.2021). Patient data were anonymized to ensure confidentiality and compliance with ethical standards for medical research.

## 3 Results

### 3.1 Demographic characteristics

The comparative analysis of patients diagnosed with peritonsillar phlegmon, laterocervical/submandibular abscess, and peritonsillar abscess was conducted using demographic and clinical parameters, described in Table 1. Mean values and standard deviations were calculated for continuous variables, while categorical variables were analyzed based on percentage distributions. The statistical significance of differences between groups was assessed using *p*-values.

**TABLE 1:** Demographic and clinical characteristics of patients with peritonsillar and cervical infections.

Parameters		Groups						Cohort		*p*
		I		II		III				
		N	%	N	%	N	%	N	%	
Age (Mean + SD)		35.13	12.26	38.91	36.79	17.51	17.80	35.89	18.02	0.694
BMI (Mean + SD)		26.28	5.04	27.80	4.10	27.31	4.76	27.39	4.55	0.683
Sex	Feminin	5	62.5	11	32.4	30	45.5	46	42.6	0.219
	Masculin	3	37.5	23	67.6	36	54.5	61	57.4	
Environment	Rural	3	37.5	25	73.5	28	42.4	56	51.9	0.010
	Urban	5	62.5	9	26.5	38	57.6	51	48.1	

SD, standard deviation; BMI, body mass index; N, number of patients; p, statistically significance.

The mean age of patients varied across groups: 35.13 years for those with peritonsillar phlegmon, 38.91 years for those with laterocervical/submandibular abscess, and 35.89 years for those with peritonsillar abscess. The differences between the groups were not statistically significant (*p* = 0.694), indicating that age does not significantly influence the type of infection developed.

BMI values were similar across the groups, ranging from 26.28 ± 5.04 in patients with peritonsillar abscess to 27.80 ± 4.10 in those with laterocervical/submandibular abscess. Patients with peritonsillar phlegmon had a BMI of 27.31 ± 4.76. The differences were not statistically significant (*p* = 0.683), suggesting that BMI does not have a significant impact on the type of infection.

Gender analysis showed that women were more frequently affected by peritonsillar abscess (30 patients, 28%) and less frequently by peritonsillar phlegmon (5 patients, 4.7%). Among men, laterocervical/submandibular abscess was the most common diagnosis (61 patients, 57%), followed by peritonsillar abscess (35 patients, 32.7%). However, the difference was not statistically significant (*p* = 0.219).

A significant finding in the analysis was the impact of the patients’ environment of origin on the type of infection. Patients from rural areas were more frequently affected by laterocervical/submandibular abscesses (56 patients, 52.3%), while those from urban areas more commonly presented with peritonsillar abscesses (37 patients, 34.6%). This difference was statistically significant (*p* = 0.010), suggesting a potential correlation between access to dental care, oral hygiene practices, and the occurrence of these infections.

### 3.2 Dental status and oropharyngeal infections

The analysis explores the relationship between dental status and the type of oropharyngeal infection, specifically peritonsillar phlegmon, laterocervical/submandibular abscess, and peritonsillar abscess. The presence of dental work, dental implants, poor dentition, and normal dentition was examined in relation to these infections (Table 2).

**TABLE 2:** Distribution of dental status characteristics among patients with peritonsillar and cervical infections.

Parameters		Groups						Total		*P*
		I		II		III				
		N	%	N	%	N	%	N	%	
Dental work	No	4	50.0	14	41.2	26	39.4	44	40.7	0.847
	Yes	4	50.0	20	58.8	40	60.6	64	59.3	
Dental implant	No	5	62.5	24	70.6	48	72.7	77	71.3	0.830
	Yes	3	37.5	10	29.4	18	27.3	31	28.7	
Deficiency of dentition	No	6	75.0	17	50.0	37	56.1	60	55.6	0.440
	Yes	2	25.0	17	50.0	29	43.9	48	44.4	
Normal dentition	No	4	50.0	23	67.6	39	59.1	66	61.1	0.568
	Yes	4	50.0	11	32.4	27	40.9	42	38.9	

N, number of patients; p, statistically significance.

Patients with dental work were more frequently found in the peritonsillar abscess group (37%), whereas those without dental work were predominant in the laterocervical/submandibular abscess group (13%). Similarly, patients without dental implants were more prevalent across all groups, particularly in the peritonsillar abscess group (44.4%). However, the Kruskal-Wallis statistical test indicated no significant association (*p* > 0.05), suggesting that dental work and implants are not determining factors in the development of a specific type of infection.

Poor dentition was observed in 26.9% of patients with peritonsillar abscess and 15.7% of those with laterocervical/submandibular abscess, but the differences between groups were not statistically significant (*p* = 0.440). Conversely, the absence of normal dentition was more common in the peritonsillar abscess group (36.1%); however, this variable also did not show statistically significant differences (*p* = 0.568).

These findings suggest that while poor dental health is prevalent among patients with oropharyngeal infections, no single aspect of dental status appears to significantly influence the type of infection developed.

**FIGURE 1:**
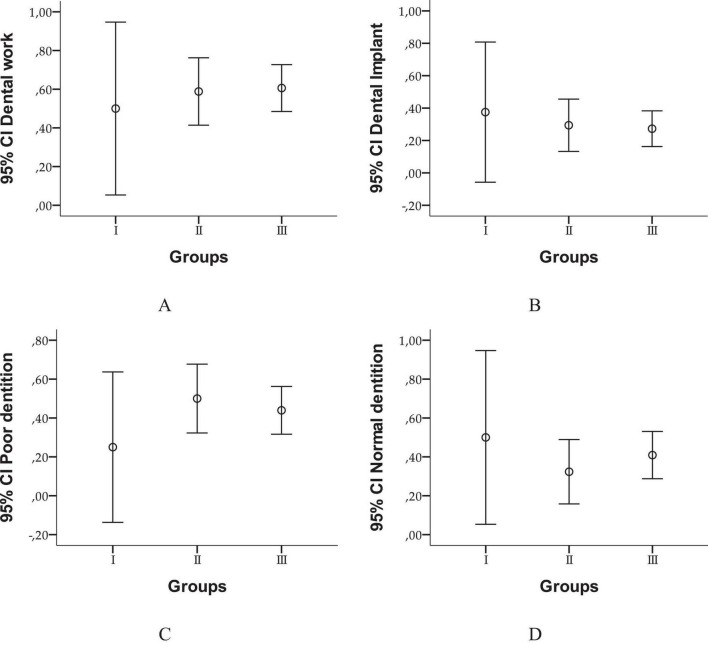
95% Confidence intervals for dental status across study groups. This figure presents error bar plots illustrating the distribution of dental work **(A)**, dental implants **(B)**, poor dentition **(C)**, and normal dentition **(D)** across the peritonsillar phlegmon, laterocervical/submandibular abscess, and peritonsillar abscess groups.

Although patients with peritonsillar abscesses appear to have more frequent dental issues, statistical tests do not confirm a significant association between dental status and the type of infection (Figure 1). This suggests that other factors, such as oral hygiene or comorbidities, may have a greater impact.

### 3.3 Associated comorbidities

Table 3 presents the distribution of patients across the three study groups based on the presence of comorbidities, including diabetes mellitus (DM), hypertension (HTN), chronic heart failure (CHF), acute myocardial infarction (AMI), stroke (SCI), and liver diseases. It also includes the statistical significance (*p*-value) to determine whether differences between the groups are statistically relevant.

**TABLE 3:** Distribution of comorbidities among patients with peritonsillar and cervical infections.

Parameters		Groups						Total		*P*
		I		II		III				
		N	%	N	%	N	%	N	%	
DM	No	6	75.0	32	94.1	61	92.4	99	91.7	0.149
	Yes	2	25.0	2	5.9	5	7.6	9	8.3	
HTN	No	8	100.0	26	76.5	56	84.8	90	83.3	0.206
	Yes	0	0.0	8	23.5	10	15.2	18	16.6	
CHF	No	8	100.0	32	94.1	60	90.9	100	92.6	0.702
	Yes	0	0.0	2	5.9	6	9.1	8	7.4	
AMI	No	8	100.0	33	97.0	62	93.9	103	95.4	0.780
	Yes	0	0.0	1	3.0	4	6.1	5	4.6	
SCI	No	8	100.0	34	100.0	64	96.7	106	98.1	0.728
	Yes	0	0.0	0	0.0	2	3.3	2	1.9	
Liver diseases	No	7	87.5	32	94.1	61	92.4	100	92.6	0.782
	Yes	1	12.5	2	5.9	5	7.6	8	7.4	

DM, Diabetes mellitus; HTN, hypertension; CHF, chronic heart failure; AMI, acute myocardial infarction; SCI, stroke and liver diseases; N, number of patients; p, statistical significance.

Regarding diabetes mellitus (DM), the majority of patients (92.5%) do not have this condition, while only 7.5% are diagnosed with DM. The distribution of diabetic patients is relatively balanced across the three groups, with no statistically significant difference (*p* = 0.149), suggesting that DM does not significantly influence the likelihood of developing a particular type of infection.

For hypertension (HTN), 15.9% of patients were hypertensive, with most cases found in Group 2 and Group 3. However, the majority of patients (84.1%) did not have this comorbidity. The differences between groups were not statistically significant (*p* = 0.206), indicating that HTN is not directly associated with any specific infection type.

In the case of chronic heart failure (CHF), only 6.5% of patients had this condition, with a slightly higher prevalence in Group 3. However, the statistical test showed no significant difference (*p* = 0.702), indicating that CHF does not have a clear association with any of the infection types analyzed.

For acute myocardial infarction (AMI), 3.7% of patients had a history of AMI, with a higher proportion in Group 3. However, there was no statistically significant difference between groups (*p* = 0.780), suggesting that a history of myocardial infarction does not significantly influence the distribution of patients among the infection groups.

Regarding stroke (SCI), only one patient (0.9%) had a history of stroke, indicating a very low prevalence of this condition within the study sample. The statistical test (*p* = 0.728) confirmed that stroke does not have a significant impact on the distribution of patients across the infection groups.

For liver diseases, 6.5% of patients were diagnosed with this condition, with most cases found in Group 3. However, the statistical test indicated no significant association (*p* = 0.782), suggesting that liver diseases do not play a determining role in the type of infection developed. These findings suggest that while certain comorbidities, such as hypertension and liver disease, are more prevalent in specific groups, none of the analyzed conditions showed a statistically significant association with a particular type of oropharyngeal infection.

**FIGURE 2:**
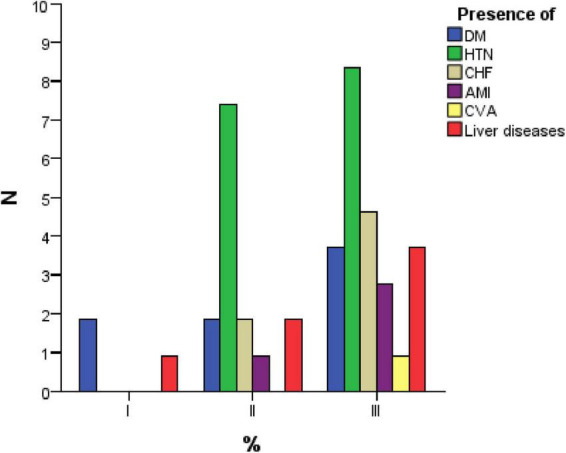
Distribution of comorbidities among study groups.

The comorbidity analysis indicates that none of the investigated conditions (DM, HTN, CHF, AMI, CVA, or liver diseases) show statistically significant differences between groups (*p* > 0.05). Although certain comorbidities, such as DM, HTN, and CHF, are more prevalent in Group 3, these differences are not substantial enough to establish a definitive correlation between comorbidities and the type of infection. Therefore, other factors, such as dental status or overall health conditions, may have a greater impact on the severity and type of infection (Figure 2).

### 3.4 Inflammatory indicators

Table 4 presents the mean values and standard deviations (SD) for two inflammatory markers, erythrocyte sedimentation rate (ESR, mm/h) and C-reactive protein (CRP, mg/dL), in patients from the three study groups: Peritonsillar phlegmon, laterocervical/submandibular abscess, and peritonsillar abscess. The *p*-value is also included to assess the statistical significance of differences between the groups.

**TABLE 4:** Comparison of inflammatory markers (ESR and CRP) among patients with peritonsillar and cervical infections.

Parameters	Groups						Total		*P*
	I		II		III				
	Mean	SD	Mean	SD	Mean	SD	Mean	SD	
ESR mm/h	40.5	22.2	35.1	24.3	43.2	28.2	40.4	26.6	0.362
CPR mg/dL	109.31	90.95	100.45	96.12	91.21	81.88	95.50	86.63	0.792

ESR, erythrocyte sedimentation rate; CRP, C-reactive protein; SD, standard deviation; p, statistical significance.

Regarding ESR, patients with peritonsillar abscess had a mean value of 43.2 mm/h ± 28.2, followed by those with peritonsillar phlegmon (40.5 mm/h ± 22.2) and laterocervical/submandibular abscess (35.1 mm/h ± 24.3). The statistical test (*p* = 0.362) showed no significant differences between the groups, indicating that ESR cannot be considered a clear differential marker between these types of infections.

For CRP, the highest mean values were observed in patients with peritonsillar phlegmon (109.31 mg/dL ± 90.95), followed by those with laterocervical/submandibular abscess (100.45 mg/dL ± 96.12) and peritonsillar abscess (91.21 mg/dL ± 81.88). However, the statistical test (*p* = 0.792) indicated that these differences were not statistically significant. This suggests that CRP levels do not vary significantly based on the type of infection and may not serve as a distinguishing factor among these conditions.

**FIGURE 3:**
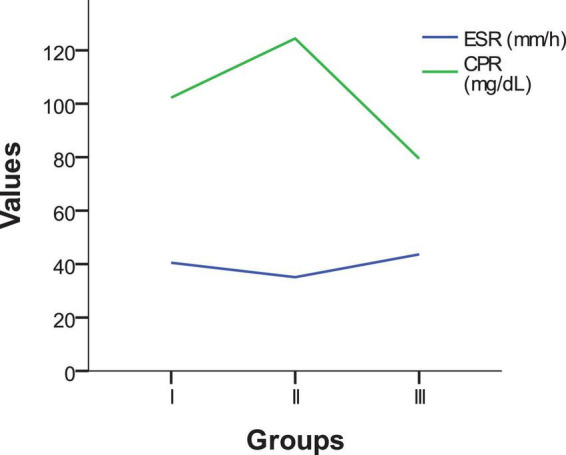
Comparison of inflammatory markers (ESR and CRP) across study groups.

The analysis of these two inflammatory markers (ESR and CRP) shows that, although variations exist between groups Figure 3, the differences are not statistically significant (*p* > 0.05). Neither ESR nor CRP can be used as definitive indicators for differentiating between the types of infections analyzed. However, the elevated levels of these markers confirm the presence of an active inflammatory process in all patients included in the study.

### 3.5 Surgical intervention and treatment characteristics

#### 3.5.1 Need for surgery: incision and drainage or other surgical methods

The need for surgical intervention varied based on dental status, with differences observed across the three infection types: Peritonsillar phlegmon, laterocervical/submandibular abscess, and peritonsillar abscess (Table 5).

**TABLE 5:** Average length of hospitalization (in days) based on dental status and type of infection.

	Groups		
Categories	I	II	III
**Dental work**			
Without dental work	7.00	7.36	6.35
With dental work	5.75	7.50	6.75
**Dental implant**			
Without dental implant	6.80	8.00	7.10
With dental implant	5.67	6.10	5.22
**Deficit dentition**			
Without deficient dentition	6.83	7.59	6.43
With deficient dentition	5.00	7.29	6.79
**Normal dentition**			
Without normal dentition	6.75	7.52	6.44
With normal dentition	6.00	7.27	6.81

Patients without dental work had a lower rate of surgical intervention, particularly for peritonsillar phlegmon (2.8%) and peritonsillar abscess (18.5%). In contrast, those with dental work required surgical procedures more frequently, especially for peritonsillar abscess (36.1%) and laterocervical/submandibular abscess (15.7%). These findings suggest that patients with dental work may have had more severe infections, necessitating more frequent interventions.

Regarding dental implants, patients without implants had the highest rate of surgical interventions, particularly for peritonsillar abscess (38.9%) and laterocervical/submandibular abscess (17.6%). Conversely, those with dental implants exhibited a lower rate of surgical procedures, with no recorded interventions for peritonsillar abscess (0.0%). This may indicate better oral hygiene or a reduced predisposition to severe infections among individuals with dental implants.

For poor dentition, patients without poor dentition had a significantly higher rate of surgical interventions for peritonsillar abscess (28.7%). In contrast, those with poor dentition required fewer interventions for peritonsillar abscess (0.0%) but had a relatively high surgical rate for peritonsillar phlegmon (25.9%). These results suggest that poor dentition may contribute to infections that require surgical intervention.

Patients without normal dentition had a high rate of surgical interventions for peritonsillar abscess (35.2%) and laterocervical/submandibular abscess (16.7%). Those with normal dentition had a lower rate of surgical interventions, suggesting that a healthy dentition may offer some protective effect against severe infections requiring surgical treatment.

These findings highlight the potential influence of dental status on infection severity and the need for surgical intervention.

Patients with dental work and without normal dentition had the highest rate of surgical intervention, suggesting that these factors may be associated with more severe infections.

Dental implants appeared to be associated with a lower rate of surgical intervention, potentially reflecting better overall oral health or a protective effect against severe infections.

Poor dentition was linked to an increased rate of surgical intervention for peritonsillar abscess, indicating that compromised dental health may contribute to the severity of infections requiring surgical management.

Conversely, normal dentition appeared to provide a protective advantage, as patients with normal teeth had a lower rate of surgical intervention for both peritonsillar and laterocervical/submandibular abscesses.

### 3.6 Length of hospital stay

Table 6 presents the mean length of hospital stay (in days) for patients diagnosed with peritonsillar phlegmon, laterocervical/submandibular abscess, and peritonsillar abscess, based on their dental status. The analysis considers the presence of dental work, dental implants, poor dentition, and normal dentition.

**TABLE 6:** Percentage distribution of patients according to treatment type and dental status.

Categories	Type of infection	Therapy		
		Antibiotic + Analgesic	Antibiotic + Analgesic + Antipyretic	Antibiotic + Analgesic + Antipyretic + Corticosteroid
**Dental work**				
Without dental work	Peritonsillar phlegmon	2.8	0.0	0.9
	Laterocervical/submandibular abscess	8.3	2.8	1.9
	Peritonsillar abscess	20.4	2.8	0.9
With dental work	Peritonsillar phlegmon	3.7	0.0	0.0
	Laterocervical/submandibular abscess	12.0	3.7	2.8
	Peritonsillar abscess	32.4	0.9	3.7
**Dental implant**				
Without dental implant	Peritonsillar phlegmon	3.7	0.0	0.9
	Laterocervical/submandibular abscess	13.0	5.6	3.7
	Peritonsillar abscess	37.0	2.8	4.6
With dental implant	Peritonsillar phlegmon	2.8	0.0	0.0
	Laterocervical/submandibular abscess	7.4	0.9	0.9
	Peritonsillar abscess	15.7	0.9	0.0
**Deficit dentition**				
Without deficient dentition	Peritonsillar phlegmon	4.6	0.0	0.9
	Laterocervical/submandibular abscess	8.3	3.7	3.7
	Peritonsillar abscess	28.7	2.8	2.8
With deficient dentition	Peritonsillar phlegmon	1.9	0.0	0.0
	Laterocervical/submandibular abscess	12.0	2.8	0.9
	Peritonsillar abscess	24.1	0.9	1.9
**Normal dentition**				
Without normal dentition	Peritonsillar phlegmon	2.8	0.0	0.9
	Laterocervical/submandibular abscess	13.9	5.6	1.9
	Peritonsillar abscess	32.4	1.9	1.9
With normal dentition	Peritonsillar phlegmon	3.7	0.0	0.0
	Laterocervical/submandibular abscess	6.5	0.9	2.8
	Peritonsillar abscess	20.4	1.9	2.8

The analysis of hospital stay duration revealed variations based on dental status; however, the differences were not statistically significant.

Patients without dental work had a mean hospital stay of 7.00 days for peritonsillar phlegmon, 7.36 days for laterocervical/submandibular abscess, and 6.35 days for peritonsillar abscess. In contrast, those with dental work had slightly shorter hospital stays for peritonsillar phlegmon (5.75 days), peritonsillar abscess (6.75 days), and laterocervical/submandibular abscess (7.50 days). These findings suggest that the presence of dental work does not significantly impact the length of hospitalization.

Patients without dental implants had the longest hospital stays, particularly for laterocervical/submandibular abscess (8.00 days), with a mean duration of 6.80 days for peritonsillar phlegmon and 7.10 days for peritonsillar abscess. Conversely, those with dental implants had shorter hospital stays across all groups: 5.67 days for peritonsillar phlegmon, 6.10 days for laterocervical/submandibular abscess, and 5.22 days for peritonsillar abscess.

Regarding poor dentition, patients without poor dentition had hospital stays of 6.83 days for peritonsillar phlegmon, 7.59 days for laterocervical/submandibular abscess, and 6.43 days for peritonsillar abscess. On the other hand, those with poor dentition had shorter hospital stays for peritonsillar phlegmon (5.00 days) but similar durations for peritonsillar abscess (6.79 days) and laterocervical/submandibular abscess (7.29 days). These findings indicate that poor dentition does not have a major impact on hospital stay duration.

Patients without normal dentition had the longest hospital stays for laterocervical/submandibular abscess (7.52 days), while those with normal dentition had shorter hospital stays, especially for peritonsillar phlegmon (6.00 days).

Overall, patients with dental implants had the shortest hospital stays, particularly for peritonsillar phlegmon (5.67 days) and peritonsillar abscess (5.22 days). Patients without dental implants had the longest hospital stays, especially for laterocervical/submandibular abscess (8.00 days). Dental work did not appear to significantly influence hospitalization duration, as the values remained similar between patients with and without dental work. Additionally, patients with normal dentition tended to recover faster, whereas those without normal dentition required longer hospitalization, particularly for laterocervical/submandibular abscess.

### 3.7 Allopathic treatment

The treatment approach varied according to dental status, particularly in terms of the administration of antibiotics, corticosteroids, and combination therapies (Table 7).

**TABLE 7:** Meta-regression summary table.

Component	Value
Effect size	Age group
Standard error	Sex
Model	Random-effects meta-regression
Method	REML
SE adjustment	None
Included cases (N, %)	62 (57.4%)
Excluded cases (N,%)	46 (42.6%)
Total cases (N,%)	108 (100.0%)
Chi-square (Q)	53.211
Degrees of freedom (df)	55
*p*-value (Sig.)	0.543
Tau-squared	0.000
I-squared (%)	0.0
H-squared	1.000
R-squared (%)	100.0

Patients with dental work more frequently received complex treatments, including antibiotic + analgesic + antipyretic + corticosteroid therapy, especially in cases of peritonsillar abscess (3.7%). They also had a higher frequency of antibiotic + analgesic therapy alone, particularly for peritonsillar abscess (32.4%). In contrast, patients without dental work received corticosteroid therapy less frequently.

Patients without dental implants had the highest rate of antibiotic + analgesic therapy alone, particularly for peritonsillar abscess (37.0%). On the other hand, patients with dental implants had lower rates of corticosteroid administration, which may indicate a more favorable course of infection in this group.

For poor dentition, patients with poor dentition received corticosteroid therapy less frequently but were more commonly treated with antibiotic + analgesic therapy, especially for peritonsillar abscess (24.1%) and laterocervical/submandibular abscess (12.0%). In contrast, patients without poor dentition had a higher frequency of corticosteroid therapy, suggesting a potential correlation between poor dentition and a milder progression of the infection.

Patients without normal dentition more frequently received exclusive antibiotic + analgesic therapy, particularly for peritonsillar abscess (32.4%). Those with normal dentition had a higher frequency of corticosteroid therapy (2.8% for peritonsillar abscess), which may indicate a more severe infection course in this group.

Overall, patients with dental work and without normal dentition received more frequent and complex treatments, including corticosteroid therapy, suggesting a more severe infection progression. Patients without dental implants had the highest rate of antibiotic and analgesic administration, potentially reflecting more extensive inflammation. In contrast, patients with poor dentition received less aggressive treatments, which may indicate either a less severe infection or a different treatment response. Additionally, patients without normal dentition required corticosteroid therapy more frequently, which could suggest a more severe inflammatory response in these cases.

### 3.8 Meta regression study

The meta-regression model (Table 8), employing a random-effects framework with restricted maximum likelihood (REML) estimation, explored the relationship between age category (Categ_varsta) as the effect size and sex as the standard error moderator. The model included 62 out of 108 total cases (57.4%), with 46 excluded due to missing or incomplete data.

**TABLE 8:** Percentage of patients requiring surgical intervention by dental status and type of infection.

Dental status category	Phlegmon (%)	Cervical abscess (%)	Peritonsillar abscess (%)
Dental work: Absent	2.8	3.0	5.0
Dental work: Present	0.0	15.7	36.1
Dental implants: Absent	3.7	17.6	38.9
Dental implants: Present	2.8	7.4	15.7
Poor dentition: Absent	1.9	3.7	0.9
Poor dentition: Present	0.0	12.0	25.9
Normal dentition: Present	3.7	0.9	2.8
Normal dentition: Absent	1.9	16.7	35.2

The residual heterogeneity test yielded a non-significant Q statistic (Q = 53.211, df = 55, *p* = 0.543), suggesting no meaningful unexplained variability beyond what would be expected by chance. Consistently, the tau-squared estimate was 0.000, and the I-squared value was 0%, indicating a lack of observed heterogeneity across studies. The H-squared value was 1.000, supporting the conclusion that the observed variability does not exceed expected sampling variation. Notably, the R-squared value of 100% suggests that the included moderator (sex) accounts for all explainable between-study variance under the model.

These findings imply that, within the limitations of this dataset, there is no significant heterogeneity in effect sizes, and the moderator variable (sex) effectively explains the observed variance in the model. However, given the modest sample size and exclusion rate, these conclusions should be interpreted with caution.

**FIGURE 4:**
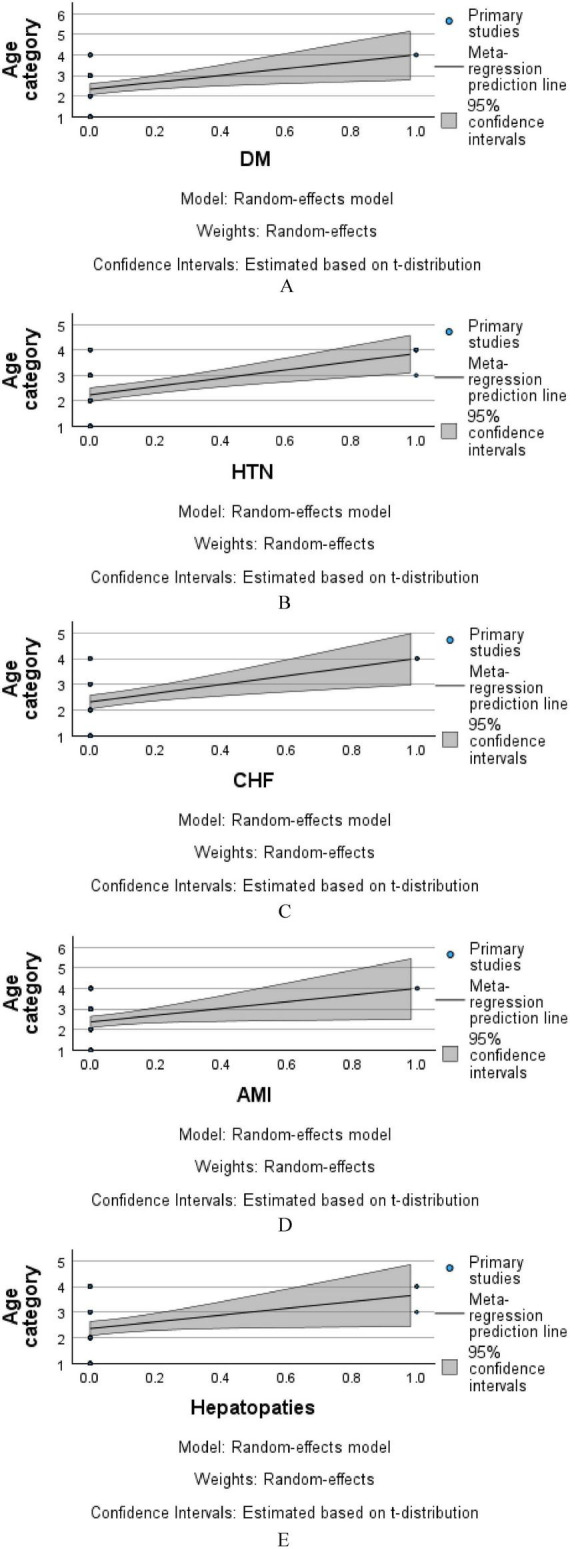
Meta-regression analysis of age category by comorbidity **(A–E)**.

Figure 4 illustrates the meta-regression analysis evaluating the association between age category and the presence of selected comorbidities, using a random-effects model. In each panel, the *x*-axis represents the presence or absence of a comorbidity (coded from 0 to 1), while the y-axis denotes age category. Figure 4A (DM—Diabetes Mellitus): A slight positive trend was observed between diabetes and higher age categories, suggesting that diabetic patients in this cohort tended to be older. However, the wide 95% confidence interval around the regression line indicates limited precision and a lack of strong statistical association. Figure 4B (HTN—Hypertension): A similar positive slope is observed, indicating that hypertensive patients are more likely to fall into higher age categories. Again, the confidence interval is relatively broad, suggesting the effect is not statistically robust. Figure 4C (CHF—Chronic Heart Failure): The regression line indicates a modest positive relationship, with older age groups showing a slightly increased prevalence of CHF. However, the overlapping confidence interval suggests this trend is not statistically significant. Figure 4D (AMI—Acute Myocardial Infarction): The trend line shows an upward trajectory, implying a potential association between AMI and older age. Yet, due to the small number of patients with AMI, the model remains underpowered to confirm a strong relationship. Figure 4E (Hepatopathies): A slight positive correlation is observed between hepatopathy presence and higher age categories. As with other comorbidities, the wide confidence band highlights the need for cautious interpretation.

Collectively, these plots suggest that while certain comorbidities appear more common in older age categories within the sample, none of the associations reached statistical significance. The broad confidence intervals reflect the modest sample size and variability within groups. Further prospective analysis with larger cohorts is warranted to confirm age-related trends in comorbidity distribution.

## 4 Discussion

Our study demonstrated that patients with dental work and no normal dentition required more aggressive treatments, including corticosteroid therapy, suggesting a more severe course of infection. This observation aligns with existing literature, which indicates that dental infections can progress rapidly, leading to significant functional impairments such as respiratory and swallowing difficulties (13, 14).

Additionally, our findings show that patients with dental work had a higher incidence of corticosteroid therapy use. Although we did not identify specific studies directly correlating dental work with corticosteroid therapy in oropharyngeal infections, the literature suggests that dentoalveolar inflammatory processes may be risk factors for deep cervical space infections, often necessitating more intensive therapeutic interventions (15–17).

Our study highlights the potential benefits of monitoring oral health and preventing dental conditions to reduce the severity of peritonsillar and cervical infections. This conclusion is consistent with previous research emphasizing timely prevention and treatment of dental infections to mitigate severe complications (13, 18, 19).

Peritonsillar and cervical infections, such as peritonsillar phlegmon and laterocervical or submandibular abscess, represent severe complications of oropharyngeal infections. Recent studies report an increasing incidence of peritonsillar abscess, which may be partly attributed to rising bacterial resistance to standard treatments for tonsillitis (20–22).

These infections have significant epidemiological implications, as they can spread rapidly and cause systemic complications, often requiring urgent medical and surgical interventions. The correlation between oral health and the occurrence of these infections is well-documented; poor oral hygiene and untreated dental conditions serve as foci of infection, facilitating bacterial dissemination to peritonsillar and cervical structures (23–28).

Maintaining good oral hygiene and promptly treating dental conditions not only prevents localized complications but also has a broader public health impact, reducing the incidence and severity of peritonsillar and cervical infections (29–32).

### Limitations

One of the primary limitations of this study is the relatively small sample size, which may impact the generalizability of the findings. A larger patient cohort would allow for a more robust statistical analysis, reducing individual variability. Additionally, the retrospective nature of the study, which relied on data extracted from medical records, may introduce biases related to data completeness and the inability to control all confounding factors.

Another limitation is the lack of direct oral hygiene assessment. While the study evaluated dental status (presence of implants, dental work, and poor dentition), objective oral hygiene indicators—such as bacterial plaque scores or periodontal disease status—were not included. This limitation prevents a comprehensive understanding of the relationship between oral health and infection severity.

Additionally, other confounding factors such as comorbidities, long-term medication use, lifestyle habits (smoking, alcohol consumption, and diet), and access to dental care were not extensively detailed in the analysis, even though they may influence infection severity and treatment response. Furthermore, the study only focused on hospitalized patients, without assessing the long-term impact of dental status on infection recurrence or late complications.

### Perspectives for future research

To confirm and expand our findings, prospective studies with larger sample sizes are necessary to further investigate the relationship between dental status and oropharyngeal infection severity. Future studies should include objective oral hygiene parameters, such as bacterial plaque index, gingival bleeding, and adherence to dental prophylaxis techniques.

An additional research focus should be the correlation between inflammatory markers (CRP, ESR, leukocyte count) and dental status, to determine whether patients with dental disease exhibit higher levels of systemic inflammation. Another critical area of investigation is the impact of early dental treatment on the progression of peritonsillar and cervical infections, to assess whether preventive dental care can lower infection incidence and severity.

An interdisciplinary approach, involving ENT specialists, dentists, and infectious disease experts, could help develop more effective prevention and treatment protocols, ultimately reducing the need for surgical interventions and hospital stays. Future research in this field could significantly improve therapeutic strategies and preventive measures for oropharyngeal infections associated with dental health.

## 5 Conclusion

Our findings suggest that dental status may be associated with the severity and treatment complexity of peritonsillar and cervical infections. Specifically, patients with prior dental work and non-intact dentition were more likely to require corticosteroid therapy and surgical interventions. Patients without dental implants experienced longer hospital stays and more frequent antibiotic administration, possibly indicating more intense inflammation.

Although these findings do not establish causation, they highlight potential relationships between oral health indicators and infection outcomes. Poor dentition, while not directly linked to more aggressive treatment, was observed in patients with more severe infection types. These associations underscore the importance of routine oral health monitoring and timely dental care as potential strategies to mitigate the burden of oropharyngeal infections.

Future prospective research incorporating detailed oral hygiene assessments and microbial profiling is warranted to further investigate these relationships and to determine whether early dental interventions can modify the clinical trajectory of these infections.

## Data Availability

The original contributions presented in the study are included in the article/supplementary material, further inquiries can be directed to the corresponding author.
